# Translation and cultural adaptation into Brazilian culture of type 1 diabetes distress scale

**DOI:** 10.1186/s13098-017-0260-y

**Published:** 2017-08-07

**Authors:** M. S. V. M. Silveira, T. G. Bovi, P. F. Oliveira, E. J. Pavin, L. Fisher

**Affiliations:** 1 Internal Medicine Postgraduate Program, Faculty of Medical Sciences-Unicamp, Campinas, Brazil; 2 Statistical Research Center, Faculty of Medical Sciences-Unicamp, Campinas, Brazil; 3 Endocrinology Division, Department of Internal Medicine, Faculty of Medical Sciences-Unicamp, Rua Vital Brasil, 251, Barão Geraldo, Campinas, São Paulo CEP: 13083970 Brazil; 4 Department of Family and Community Medicine, University of California San Francisco-UCSF, San Francisco, USA

**Keywords:** Diabetes mellitus type 1, Emotional distress, Translating

## Abstract

**Background:**

Diabetes related distress is common in type 1 *diabetes* patients (T1D). High levels of *diabetes distress* are related to poor metabolic control. An instrument to evaluate diabetes distress in T1D patients is “type 1 diabetes scale-T1DDS”. The aim of this study was to translate and culturally adapt the T1DDS into Brazilian culture.

**Methods:**

T1DDS scale was translated into Portuguese. Back translation was performed and evaluated by a specialists committee. Pre-test was performed with 40 T1D outpatients at State University of Campinas hospital. Internal consistency, external consistency and re-test were performed.

**Results:**

72% women, mean age: 32, 1 ± 9, 7 years, mean diabetes duration: 15, 8 ± 9, 1 years, mean scholarity: 11, 5 ± 3, 6, glycosylated hemoglobin mean: 9 ± 2%. Internal consistency: Cronbach alpha of T1DDS Brazilian version was 0.93. External consistency: Spearman’s coefficient between T1DDS and PAID, Brazilian version, was 0.7781; (p < 0.0001).

**Conclusions:**

The T1DDS Brazilian version is a reliable tool to evaluate *diabetes distress* in T1D patients in the Brazilian Population. This tool can be useful in clinical care and to identify patiens at risk and in need for psychosocial intervention.

## Background

An important focus of type 1 diabetes (T1D) treatment is to keep blood sugar level within a target range and to avoid glycemic variability. Uncontrolled hyperglycemia over time is associated with the development of micro and macrovascular complications [1].

To achieve these goals, T1D patients need to, monitor their blood sugar frequently and regularly, and address the effects of carbohydrate consumption and exercise to keep levels of blood sugar at acceptable levels.

Living with T1D, the demands of glycemic control and dealing with the possibility of developing complications combine to increase diabetes-related distress (DD). High DD has been shown to reduce self management and affect glycemic control [2–5]. Concerns about emotional and behavioral aspects related to metabolic control, remain challenging to researchers and clinicians.

High DD has been shown to be quite prevalent among T1D adults [6–8]. DD may be defined as a group of reactions and emotional responses to life with diabetes, specifically associated with treatment, diet and self-management demands. It is related to sadness, frustration, anger, disappointment, fatigue, disorganization and “burnout” related to diabetes management [9] and it is the result of personal experiences of people dealing with diabetes [9]. DD is distinct from clinical depression and is directly linked to poor glycemic control [10].

DD may hamper the ability of patients to manage their disease and reach treatment goals. Studies have shown the importance of correctly diagnosing DD to help develop strategies aimed at efficient management of disease in clinical practice [7, 11].

An important tool to evaluate DD is the “type 1 diabetes distress scale”—T1DDS, that addresses the distress associated with self-monitoring, insulin adjustment, fears about hypoglycemia, family and social perceptions of disease, among others [12].

The T1DDS was developed in the USA, by Fisher et al. [12] following a qualitative study that reported the high prevalence of DD and the common sources of DD in this population, e.g., feelings of powerlessness, concerns about management, fears of hypoglycemia, etc. [13]. Initial potential items for T1DDS were developed by the authors in consultation with adults with T1D and diabetes health care professionals.

Over 50 items were originally identified and they were administered to a large sample of T1D patients. Exploratory and confirmatory factor analyses reduced the number of items and demonstrated item clusters that formed individual subscales. T1DDS differs from other previously applied scales for proposing questions more related to T1D patients lives.

To utilize the T1-DDS in other countries and with other languages, translation and cross cultural adaptation are required. Brazil is a country with considerable social and cultural diversity, including families of different incomes, health care and food choices [14]. Thus, the aim of this study was to translate and adapt the T1-DDS for use with T1D adults in Brazil.

## Methods

This study was performed according to proceedings for instruments translation and transcultural validation [14].

### Subjects

40 T1D patients followed at type 1 diabetes clinic of the State University of Campinas hospital, a tertiary university hospital, in Campinas, São Paulo, Brazil, were evaluated, between September 2016 and February 2017. They were invited to take part on the study during routine consultations.

Inclusion criteria were: age 18 years and older with diagnosis of T1D for at least 6 months. Exclusion criteria were cognitive impairment that could harm the patients’ ability to answer the scale questions, history of major psychiatric disorders (such as schizophrenia, drug addiction, dementia) and patients with severe diabetes-related complications (blindness, need for hemodialysis, major limb amputations and stroke).

### Procedure

**Fig. 1:**
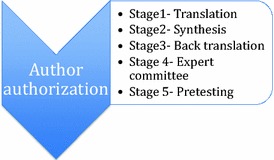
Process to translate and adapt instruments according literature

The translation and cultural adaptation followed the steps suggested by Guillemin et al. [14]. All the steps were carefully taken into account to produce a scale capable of measuring the same phenomena as the original scale, without losing its relevance or reliability (Fig. 1).

After authorization from original author, the translation of T1DDS was made by two qualified professionals fluent in English, whose native language is Brazilian Portuguese. One professional had clinical experience with DD and the other did not. The second translator was advised to carry out a semantic translation and not just a literal one, as well as to modify the text to include language appropriate for Brazilian culture.

This process resulted in two forward translations: versions 1 (T1) and 2 (T2). Where differences occurred, the translators discussed both translations and a consensus resulted in a reconciled version (T1, 2). This step was called synthesis. Subsequently, the reconciled version was translated into English by two English language native professionals, resulting in back translation (back translation BT1 and BT2). These back translations were discussed with the original author and compared to the original text to correct discrepancies.

The final version was reviewed by three endocrinologists, one psychiatrist, one nutritionist, and one T1 patient. Differences in this committee’s review were resolved by consensus.

The final translated scale was then administered to 40 adults with T1D recruited from type 1 diabetes clinic of the State University of Campinas hospital.

Cronbach was performed to evaluate the internal consistency of the scale.

Concurrent validity was evaluated by comparing T1DDS—Brazilian version to other diabetes distress scale validated in Brazilian culture—problem areas in diabetes—Brazilian version—B-PAID [15]. Twenty-five of these patients were completed a re-test of the scale 1–3 weeks later to evaluate test re-test reliability.

## Measures

Demographic measures included age, gender, education (years), marital status, income, time of disease (years) and age at diagnosis. Diabetes status included clinic-recorded HbA1c, within 3 months.

The T1-DDS has 28 items and utilizes a 6 point-likert scale, in which the patient circles a number to indicate the degree to which this is or is not a problem for them, from “not a problem” to a “serious problem.”

The original scale has 7 subscales: powerlessness (5 items), management distress (4 items), hypoglycemia distress (4 items), negative social perceptions (4 items), eating distress (3 items), physician distress (4 items), and friend/family distress (4 items).

*Powerlessness* could be described as a sense of feeling discouraged about diabetes. Example: “feeling that no matter how hard I try with my diabetes, it will never be good enough” [12].

*Negative social perceptions* refer to concerns about possible negative judgments of others. Example: “feeling that people treat me differently when they find out I have diabetes” [12].

*Physician distress* is related to disappointment with current health care professionals. Example: “feeling that I can’t tell my diabetes doctor what is really on my mind” [12].

*Friend/family distress* considers there is too much focus on diabetes amongst loved ones. Example: “feeling that my friends or family act like “diabetes police” (bother me too much)” [12].

*Hypoglycemia distress* concerns about severe hypoglycemic events. Example: “I can’t ever be safe from the possibility of a serious hypoglycemic event” [12].

*Management distress* is about disappointment with one’s own self-care efforts. Example: “I don’t give my diabetes as much attention as I probably should” [12].

*Eating distress* concerns that one’s eating is out of control. Example: “feeling that I don’t eat as carefully as I probably should” [12].

Alpha coefficients of original scale indicated good total scale reliability (total scale = 0.91, sub scale range 0.76–0.88), and 9-month test–retest reliability was excellent (total scale *r* = 0.74) [12].

## Statistics analyses

Descriptive analyses were done with measures of mean values for numerical variables and frequency (percentage) for categorical variables.

The relation between two numerical variables was measured by Spearman’s correlation coefficient.

To evaluate test/re-test confidence interval inter class was used (ICC) [16].

Internal consistency was performed by Cronbach alpha [17].

All analyses were done with SAS version 9.2 for Windows [18]. Statistical significance was 0.05.

## Results

Of all patients, 72.5% were women, 42,5% were married, 67,5% had income bellow 3 Brazilian minimum wage and mean HbA1c was 9%. The demographic, clinical and laboratorial characteristics of T1D patients are summarized in Table 1.

**Table 1:** Clinical and laboratory characteristics of T1D patients

Variable	N	Mean	Med	S.D.	Min	Max
Age	39	32.18	31.00	9.76	19.00	55.00
Scholarity	39	11.59	12.00	3.63	2.00	12.00
Time of disease	39	15.87	14.00	9.18	1.00	37.00
Age of onset	39	14.85	14.00	7.79	1.00	35.00
HbA1c	40	0.09	0.09	0.02	0.06	0.14
B-PAID-score	39	32.77	28.00	19.31	0.00	75.00
B-T1DDS-score	40	69.18	63.00	29.09	33.00	162.00

*HbA1C* glycosilated hemoglobin; *B-PAID score* problem areas in diabetes, Brazilian version score; *B-T1DDS score* type 1 diabetes distress scale, Brazilian version score

During the back translation the word “slight” (“a slight problem” in the original scale) was changed by “a little problem”.

During evaluation of specialists committee, a few words were suggested to be modified: on question 1 the word “managing” was changed by “taking care”. On question 5, the word “numbers” was changed by “values”. On question 8 the expression “much as” was changed by “quantity” and on question 21, “managing” was changed by “controlling”.

The Cronbach alpha of T1DDS Brazilian version was 0.93. The Cronbach alpha of subscales ranges from 0.61 to 0.84. The results are summarized in Table 2.

**Table 2:** T1DDS-Cronbach alpha of total scale and subscales

Subscales	Number of items	Alpha
Powerlessness	5	0.83
Negative social perception	4	0.81
Physician distress	4	0.61
Friend/family distress	4	0.84
Hypoglycemia distress	4	0.70
Management distress	4	0.70
Eating distress	3	0.83
Total distress	28	0.93

T1DDS subscales, number of items of each subscales and Cronbach alpha of total scale and each subscales

**Fig. 2:**
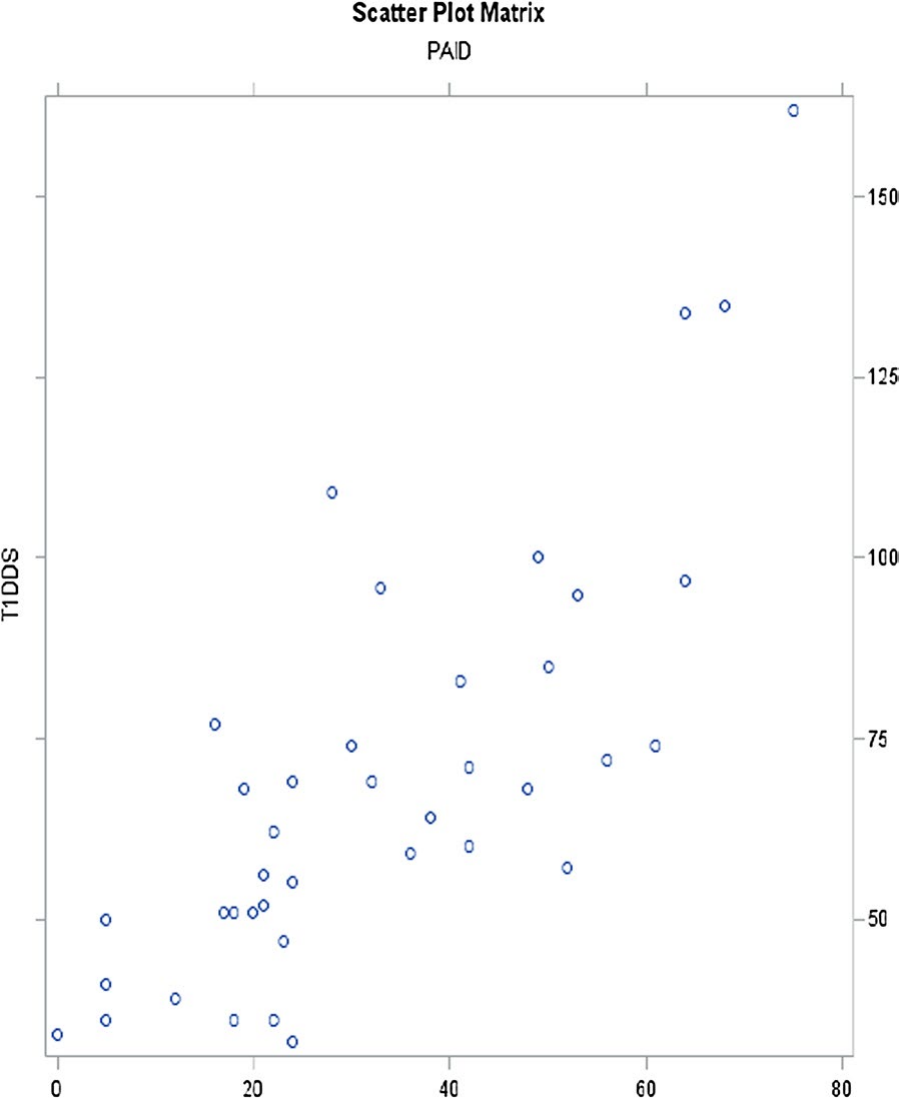
*Scatter plot matrix* relation between type 1 diabetes distress scale-T1DDS and problemas areas in diabetes-PAID

The Spearman’s coefficient between T1DDS, Brazilian version and B-PAID, Brazilian version was (0.7781); (**p < 0.0001**) (Fig. 2).

Test re-test: total-ICC: 0.94 (0.87–0.97) (ICC: 95%). The results are summarized in Table 3.

**Table 3:** T1DDS-relation between test and retest (ICC-95%)

Subscales		ICC
1	Powerlessness	0.86 (0.72–0.93)
2	Negative social perception	0.67 (0.44–0.85)
3	Physician distress	0.84 (0.69–0.92)
4	Friend/family distress	0.95 (0.90–0.98)
5	Hypoglycemia distress	0.82 (0.65–0.92)
6	Management distress	0.60 (0.35–0.81)
7	Eating distress	0.87 (0.75–0.94)
Total	Total distress	0.94 (0.87–0.97)

Relation between test and retest of T1DDS scale and each subscale

*ICC 95%* intraclass correlation coefficient

Subscale 1: high concordance; subscale 2: moderate concordance; subscale 3: high concordance; subscale 4: high concordance; subscale 5: high concordance; subscale 6: moderate concordance; subscale 7: high concordance; total scale: high concordance

### Potential for improvement

The T1 DDS, translated and adapted to Brazilian culture could be useful in clinical care to evaluate diabetes related distress in Brazilian population.

This tool could be helpful to identify patiens at risk for emotional distress who should be prioritized in psychosocial intervention.

This scale might be used in clinical research, allowing data to be collected in a padronized and trustable way.

## Discussion

Given the clinical importance of DD in the ongoing care of adults with T1D, we translated and culturally adapted the original English version of the T1-DDS into Brazilian Portuguese. We used a systematic and comprehensive set of procedures to insure adequate translation that was adapted for use in Brazilian culture.

The translated and adapted scale was considered understandable and relevant, and required only a short time period for administration.

Furthermore, it displayed adequate psychometric properties and stability over time.

Current literature suggests the importance of addressing DD in clinical care for adults with T1D [7, 11] because DD has been shown to be significantly related to disease management and glycemic control [10, 12]. We view DD not as a co-morbidity of T1D, but, like Fisher et al. [19], as an expected part of living with T1D over time. Clinically, according to Fisher et al., the T1DDS can be used to start a clinical conversation between provider and patient to acknowledge the presence of emotional distress, describe it, verbalize it, normalize it, and seek active ways of addressing it [20]. So the scale can be used not only to assess DD in these patients, but also as a clinical tool to start the process of addressing it.

In Brazil, almost 70% of T1D patients with low income receive their care in public tertiary hospitals [21]. These patients experience considerable socio-economic stress and have few community resources to help them manage their disease. For patients with low income, the demands for good managing of T1D are even more challenging and, at times, distressing. These patients frequently do not have access to modern tools and adequate professional support to help them to manage their disease so that high DD may be even more prevalent in this population than has been reported elsewhere. Consequently, we see DD as an important part of an educational and clinical program for T1D patients to help them deal with the ongoing demands of diabetes management in a resource-limited environment [10, 21].

We hope that this study might contribute to help planning psychological approaches to Brazilian T1D patients to cope and manage their disease.

## Study limitations

Study limitations include the following. First, it was conducted at a single diabetes clinic in a tertiary care hospital. Hence, it may not reflect the experience of the larger Brazilian T1D population. Second, Brazil includes several different cultural groups that may require additional scale adaptations. Third, replication with a larger sample would be helpful.

## Conclusions

Translation and cultural adaptation of T1DDS into Brazilian culture was undertaken to allow for use of the scale within the Brazilian T1D population.

The T1DDS Brazilian version is a reliable tool to evaluate DD in type1patients in Brazilian Culture.

## Abbreviations

T1DDS: type 1 diabetes distress scale
T1D: type 1 diabetes
DD: diabetes distress
B-PAID: problem areas in diabetes, Brazilian version
ICC: confidence interval inter class
T2D: type 2 diabetes
